# Sex Genotyping of Archival Fixed and Immunolabeled Guinea Pig Cochleas

**DOI:** 10.1038/s41598-018-23491-3

**Published:** 2018-03-26

**Authors:** Frédéric F. Depreux, Lyubov Czech, Donna S. Whitlon

**Affiliations:** 1 Department of Otolaryngology-Head and Neck Surgery, Northwestern University, Chicago, IL 60611 USA; 2 Interdepartmental Neurosciences Program, Northwestern University, Chicago, IL 60611 USA; 3 Knowles Hearing Center, Northwestern University, Evanston, IL 60208 USA

## Abstract

For decades, outbred guinea pigs (GP) have been used as research models. Various past research studies using guinea pigs used measures that, unknown at the time, may be sex-dependent, but from which today, archival tissues may be all that remain. We aimed to provide a protocol for sex-typing archival guinea pig tissue, whereby past experiments could be re-evaluated for sex effects. No PCR sex-genotyping protocols existed for GP. We found that published sequence of the GP *Sry* gene differed from that in two separate GP stocks. We used sequences from other species to deduce PCR primers for *Sry*. After developing a genomic DNA extraction for archival, fixed, decalcified, immunolabeled, guinea pig cochlear half-turns, we used a multiplex assay (Y-specific *Sry;* X-specific *Dystrophin*) to assign sex to tissue as old as 3 years. This procedure should allow reevaluation of prior guinea pig studies in various research areas for the effects of sex on experimental outcomes.

## Introduction

Outbred guinea pigs have been used for decades in research to study, for examples, infection, drugs, cardiology, receptors and hearing^1–8^. Some of these studies use measures that, unknown at the time, may be affected by the sex of the animal^9–11^. The sex of an animal can not only alter experimental results and conclusions, but can have a significant impact on the understanding of biological mechanisms, diseases and responses to pharmaceutical interventions. In studies of human auditory responses, differences between male and female subjects have been demonstrated with several measures of hearing including otoacoustic emissions and auditory evoked potentials, and in auditory perceptual tasks^12–18^.Clinical studies have suggested that under several conditions, males have double the chance to suffer hearing loss as females^19^. Differences in hearing responses between males and females have also been observed for example in rats^20^, mice^21–23^, chinchillas^24^ and marmosets^25^. However, much is unknown regarding sex differences in auditory physiology, anatomy, drug responses, psychoacoustics. In general throughout medical research, sex effects on experimental outcomes have been so understudied that in recent years, the National Institutes of Health (USA) has begun to formally consider sex as a biological variable and has taken steps to try to balance data from male and female animals in preclinical studies^26^ . The impetus for the present study was the desire to develop an approach to re-evaluate by sex, historical auditory physiology data. To develop this procedure, we used a sampling of archival guinea pig tissue from past experiments.

In various species, when visual inspection is insufficient (for example in fetal or young postnatal animals) or from archival materials, evaluation of the sex of an individual is generally carried out by PCR amplification of X and Y chromosome specific genes^27–31^. However, for guinea pigs, although the genome is presently under deep sequencing by the Broad Institute (Cambridge, MA) (https://www.broadinstitute.org/guinea-pig/guinea-pig-genome-project), and we could find the sequence for *Dystrophin*, an X chromosome specific gene, the gene sequence data for the Y chromosome are not yet available. We did, however, find on Genbank^32^ a partial sequence for *Sry*, a Y specific gene, but we later found that sequence to be very different from the gene in separate guinea pig stocks we acquired for our laboratory from two different breeders. Hence, we could find no published reports of PCR sex genotyping of any guinea pig tissue. Here we report our successful protocol for genomic DNA extraction from archival fixed and immunolabeled cochlear half-turns, the actual partial sequence for GP *Sry* and our PCR approach to sex genotyping DNA using *Dystrophin* and *Sry* primers in a single (multiplex) PCR reaction. Although we approached this problem from the point of view of providing methodology for determining sex effects in auditory physiology, sex genotyping of guinea pigs from archival tissue should find broad relevance across the multiple disciplines that rely on guinea pigs as an animal model.

## Results

### Genomic DNA extraction

**Figure 1:**
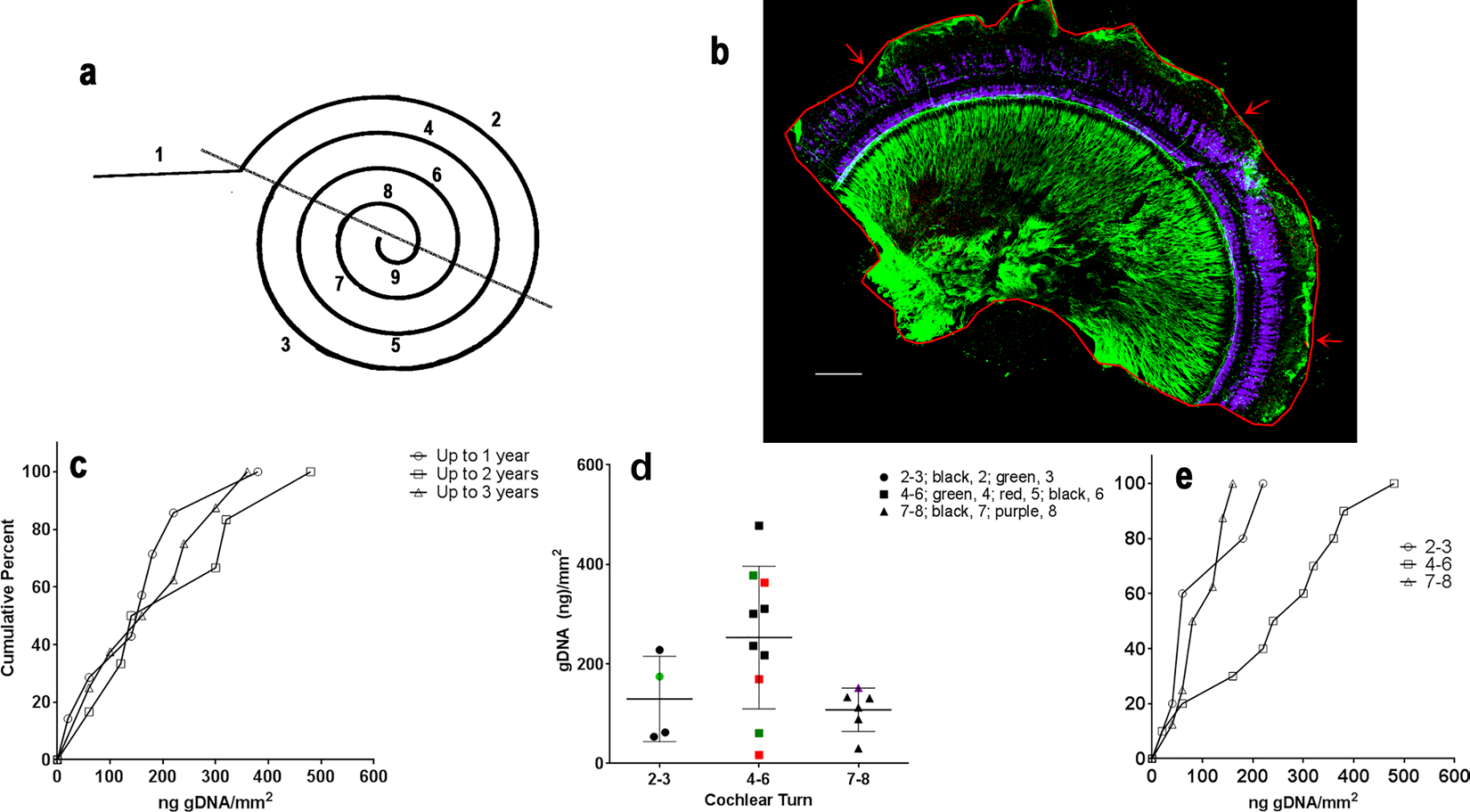
Extraction of gDNA from cochlear turns. (**a**) Diagram of the approximate locations of guinea pig cochlear turns. Number 1 is the hook region and number 9 is the apical tip. (**b**) Example of a whole mount image of an immunolabeled cochlear half turn from the mid-region. Before immunolabeling, the spiral ligament, stria vascularis and Reisner’s membranes were removed and the tissue was trimmed, reducing the thickness of the half-turn. The image is a volume reconstruction. Arrows indicate the edges of the tissue. Purple, myosin VIIa, indicates the hair cell region. Green, neurofilament, labels neurons and neurites. Green on the edges are stained non-specifically. The area of this half-turn within the red border is 0.66 mm^2^. Bar = 100 µm. (**c**) Cumulative percent histograms of gDNA/mm^2^ extracted from tissue samples stored 1, 2 and 3 years. The amount of extracted material in the sample set is similar among all years. (**d**) Combining data from turns 2–3 (base), 4–6 (mid) and 7–8 (apex) demonstrates increased variability and average extraction from the mid turns as compared to the base and apical turns. Bars represent mean ± standard deviation. (**e**) Cumulative percent histograms of gDNA extracted per area of half-turn using same data as in (**d**).

After fixation and decalcification of the bulla, the guinea pigs cochlear half-turns were dissected from the spiral cochlea. Figure 1a diagrams the approximate location of each half-turn. Turns 2–8 were used in this study. As a proof of principle, we used half-turns that had been immunolabeled, slide-mounted individually and stored at 4 °C for up to three years. For genomic DNA (gDNA) extraction, we removed one half-turn with size (area) ranging from about 0.5 mm^2^ to 2.45 mm^2^ (see example of immunolabeled cochlea half-turn Fig. 1b). From archival fixed cochleas, the half-turn surface areas averaged 1.21 ± 0.67 mm^2^. The amount of genomic DNA extracted from each half-turn varied among the samples, but averaged about 173 ± 117 ng. Because the samples were derived from past experiments used for other purposes, the thickness of the tissue was not controlled. The thicknesses were, estimated to be between 40 and 100 µm. Among all the samples, the ng of gDNA extracted per mm^2^ of tissue ranged from about 17 to 478. But the distribution of levels of gDNA amount per mm^2^ among the samples that had been stored for 1, 2 or 3 years was similar as shown by the cumulative percent histogram in Fig. 1c. The overall surface area of the half-turns decreases from base to apex (turns 2–8), from about 1.25 ± 0.544 mm^2^ to about 0.81 ± 0.319 mm^2^ with the great variability likely do to the manual dissection and may be also the outbred nature of the stock. If we group the half-turns together as base (2–3), mid (4–6), and apex (7–8), the gDNA extracted amount per mm^2^ is more variable in the mid turns than from either the base or apical turns, and the average (252.2 ± 143.5) is higher than either (129.7 ± 85.7; and 107 ± 43.7 respectively). The distribution of the values among samples for ng gDNA/mm^2^ can be seen in the cumulative percent histogram (Fig. 1e) of the same data as in Fig. 1d.

### Development and validation of guinea pig multiplex sex genotyping PCR assay

**Figure 2:**
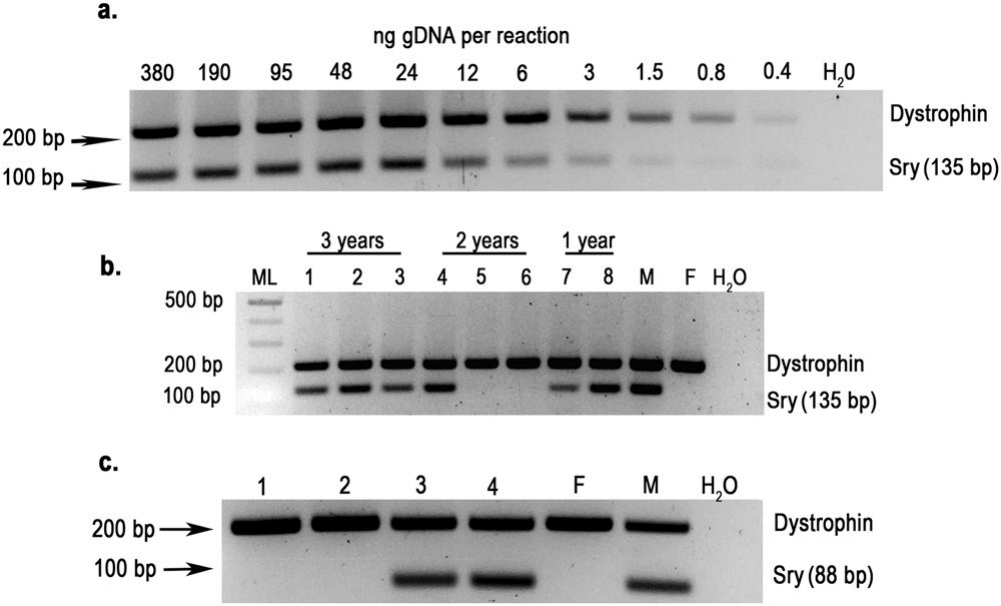
Analytical gels resolving sex genotyping multiplex PCR of guinea pig tissue. (**a**) Concentration titration of gDNA template. Representative gel (one of triplicate experiments) of PCR products from gDNA of fixed guinea pig male pinna tissue. *Dystrophin* and *Sry* PCR products are expected to run at 212 and 135 bp respectively. Negative control, water. (**b**) Sex genotyping using a first deduced guinea pig *Sry* primer set generating a 135 bp amplicon from three (lanes 1–3), two (lanes 4–6) and one (lane 7–8) year old immunostained, archived, cochlear half turns. ML, molecular ladder. M and F are control (known) male and female genomic DNA from fixed pinna extraction. Dystrophin, 212 bp. (**c**) Sex genotyping PCR using a second guinea pig *Sry* PCR primer set, specifically derived from sequencing of the original PCR product (see Fig. 3), generates an 88 bp amplicon from one (lanes 1–2) and three (lanes 3–4) year old immunolabeled archived cochlear turns. M and F are control (known) male and female genomic DNA from fixed pinna extraction. Upper band running at 212 bp is the Dystrophin amplicon. Full-length original gel images are presented in Supplementary Information Figs 1–3.

**Figure 3:**
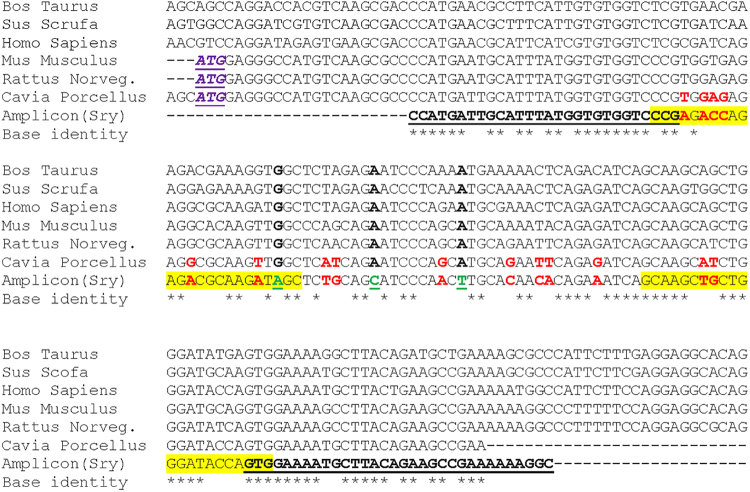
*Sry* gene sequence alignment. Guinea Pig primers for *Sry* gene were designed using *Cavia porcellus* (AJ 003126.1), *Bos taurus* (NC_016145.1), *Mus musculus* (NC_000087.7), *Rattus norvegicus* (NC_024475.1), *Homo sapiens* (NC_000024.10) and *Sus scrofa* (NC_010462.2) *Sry* sequences from Genbank. The forward and reverse sequences of the first set of *Sry* PCR primers are underlined. Rodent “ATG” start codon is in purple italic. Sequence base variations between Genbank *Cavia porcellus* (AJ003126.1) and amplicon are in red letters. (*) stars represent total base identity between all aligned sequences. Green letters are nucleotides specific to the guinea pig PCR product only. The second set of PCR primers specific to guinea pig are highlighted in yellow.

To design and validate primers for *Dystrophin* and *Sry* genes, we used the published partial GP sequences retrieved from Ensembl and GenBank databases respectively. The genomic DNA was extracted from freshly fixed pinna tissue of visually validated male and female GP donors. The PCR primers designed for *Dystrophin* (X chromosome) PCR amplification were immediately successful in generating an oligonucleotide of the expected 212 bp length (Fig. 2a). However, several attempts at creating successful primers to amplify regions of reported *Sry* gene failed, suggesting that the *Sry* in the GP stocks from two different breeders we used differed from the published sequence^32^. We then designed new primers around a *Sry* gene region that is highly conserved among a variety of other mammals (Fig. 3). The new GP primers generated a PCR product of 135 bp from male but not female guinea pig tissues (See control Male and Female lanes Fig. 2b). A multiplex PCR reaction successfully showed two bands around 200 and 100 bp for male and only one around 200 bp for female (Fig. 2a,b). To evaluate the limit of detection of the PCR sex genotyping assay, serial dilution of gDNA from fixed male pinna was performed in triplicate. The *Sry* PCR gel band was difficult to visualize below 6 ng DNA per reaction (Fig. 2a). To keep well above the limit of detection and avoid a potential source of error in sex assignment, 20 ng per reaction was routinely used with a maximum of 5 *µ*l of gDNA solution per reaction. Occasionally (less than 5% of the samples) loading between 10–20 ng was used per reaction. To confirm that each amplicon is correctly associated with the genes of interest, several independent PCR amplifications were performed on fixed female and male pinna and unfixed male liver tissues (see Materials and Methods) from the Kuiper stock and fixed cochlear tissue from the Charles River Stock. The *Dystrophin* and *Sry* amplicons were processed for Sanger sequencing. The 212 bp amplicon showed a 100% match with part of the *Dystrophin* exon 21 sequence reported by Ensembl database (Gene: ENSCPOG00000009096). The 135 bp amplicon sequence showed a good match with the published *Sry* genes of other species. Amplicon sequence analysis showed only three base pair variants (Red letters in Fig. 3) specific to our GP amplicons. However, the *Sry* amplicon sequence, identical between Kuiper and Charles River samples, showed a poorer match with the published partial GP sequence (AJ003126.1) (Fig. 3). The multiple mismatches between the published guinea pig sequence and that in the gDNA from our stocks explains our initial difficulty in designing a *Sry* primer set for this study. Chemical fixation and de-crosslinking in the procedure did not appear to have influenced the accuracy of the sequencing. The alignment of each sequencing data for Dystrophin amplicons from fixed tissue matched perfectly with the Dystrophin reference sequence (Gene: ENSCPOG00000009096; exon 21: DS562933.1:1:8140136:1) (data not shown). Similarly, we did not find any difference between sequences of *Sry* amplicons from fixed and unfixed tissues (data not shown).

### Sex genotyping of guinea pig cochlear turns

Our archival cochlear half-turns from various previous experimental protocols had originally been fixed, decalcified, immunolabeled, slide-mounted and stored at 4 °C for one, two and three years. Multiplex PCR using dystrophin (Figure b-c) and the first *Sry* guinea pig primer set (Fig. 2b) or a second *Sry* guinea pig primer set that specifically amplified the newly sequenced guinea pig *Sry* gene (Fig. 2c), successfully detected both *Dystrophin* and *Sry* genes. No notable difference in PCR quality could be observed between immunolabeled samples stored for different times.

## Discussion

Sex identification of experimental animals has become increasingly important in preclinical research, as sex effects on experimental outcomes, which might determine further clinical efficacy, are coming more to the forefront. Preclinical animal studies have a role to play in forming the questions and highlighting differences in sex effects on physiology and disease. The guinea pig has been of immense value for decades as a model for human ailments such as infection, inflammation, toxicity, cardiovascular, gastrointestinal, deafness as well as for testing drug mechanisms and effects. In the human population, sex differences are known to exist in at least some of the biological responses being modeled. For example, more men than women acquire tuberculosis^33^, vaccinations of girls and boys for diphtheria have different mortality rates^34,35^, and irritable bowel syndrome is more common in women than men^22^.

In the auditory system, sex effects on physiology and disease have not been widely examined, although effects clearly exist, as noted above. Assignment of GP sex and the effects of sex on hearing outcomes have only rarely been reported. In fact, in our perusal of 185 consecutive reports of GP auditory experiments between 2011 and 2016, we found 72 (38.9%) that did not report sex of the guinea pigs at all, 61 (33.0%) that used only one sex and 50 (27.0%) that reported using “both” sexes without further separation of results. If those studies retain appropriately archived tissue, it would be possible to return to the data and discover whether or not sexual dimorphism exists in the experimental outcomes. If one expands the thinking to other types of experiments that use guinea pigs as indicated above, it could be possible to add significant genetic information and interpretation to experiments already carried out in the past. This type of preclinical information can inform and direct future clinical studies as well as shed light on mechanisms of disease.

Reports of GP nucleic acid extraction (RNA or DNA) from archival material are very rare^36,37^. Further, we could find no protocol for processing archival fixed immunolabeled cochlear turns for any species and no reports of PCR amplification of female and male specific genes from GP.

It is likely that the lack of sex genotyping data of GP material in the literature stems from the lack of sequencing of the Y chromosome to date, and the differences we found in the sequence between our GP material and that of the published partial sequence^32^. For the Y specific *Sry* gene we choose to amplify part of the DNA binding domain HMG-box because the partial sequence was published in guinea pig^32^ and its nucleotide sequence is known to be better conserved between species^38^. Our PCR amplicon showed 18 base variants (out of 135 bp) as compared to that published for GP^32^. It is unlikely that the sequence alterations were artifacts due to fixative effects on Taq polymerase^39,40^, since two unfixed liver and three fixed pinnae from male guinea pig tissues generated the same amplicon sequence. Moreover, we did not find any base variant or insertion between *Dystrophin* 212 bp PCR product and Ensembl database cDNA sequences from similarly processed female tissues. Interestingly, at three different regions of the *Sry* gene portion, where the base sequences were conserved between the various other species aligned DNA, the new sequence of the GP Sry gene differed. Finally, the sequence we generated in the amplicon was identical between stocks derived from Kuiper and those derived from Charles River.

Overall, we aimed to validate an approach for future use of small pieces of GP cochlear turns to develop a methodology for assigning sex to prior auditory physiology experiments. For proof of principle, in our study we used a sampling of paraformaldehyde fixed, decalcified, immunolabeled cochleas from past experiments, However, any GP tissue store that can provide unadulterated genomic DNA can take advantage of the PCR primers reported here to add new knowledge to new or historic experiments and to increase understanding of sex related differences in experimental outcomes.

## Methods

### Animals

All procedures were carried out in accordance with the NIH Guide for the Care and Use of Laboratory Animals and were approved by the Institutional Animal Care and Use Committee at Northwestern University as well as the US Navy Bureau of Medicine and Surgery. Albino Hartley Guinea pigs of both sexes weighing between 390 and 863 g at the time of euthanasia were originally procured from either Kuiper Rabbit Farm (Gary, IN) or from Charles River Laboratories (Toronto, Canada) and were the source of the archived tissue samples from prior hearing experiments used in this study. For those experiments, the animals had been euthanized using barbiturate pentobarbital solution (Euthasol), then perfused intracardially with 4% paraformaldehyde (PFA) in 0.1 M Sodium Phosphate Buffer, pH 7.2. The bullas had been harvested and opened, then fixed at room temperature in 4% PFA in PBS solution for two hours. The tissue had then been washed in phosphate buffered saline (PBS) then for decalcification, placed in 10% ethylenediaminetetraacedic acid (EDTA) in PBS (pH 7.4) for 21 days, on a tissue rotator, at 4 °C, with intermittent changes of fresh EDTA solution. Tissue was stored in PBS containing 0.25% paraformaldehyde until further dissection. Fresh or immediately fixed tissues used for DNA extraction were also acquired from animals from the same breeders.

### Cochlea dissection and Immunolabeling

Cochlear tissue was originally dissected from the decalcified bullae into 9 half-turns in buffered saline as instructed by Liberman (http://www.masseyeandear.org/research/otolaryngology/investigators/laboratories/eaton-peabody-laboratories/epl-histology-resources/video-tutorial-for-cochlear-dissection). A schematic of a flattened guinea pig cochlea, indicating the approximate regions of each of the half-turns is depicted in Fig. 1a. The dissection of the cochleas into turns and the thinning of each turn for subsequent whole mount immunolabeling were carried out by hand. During the original experiments, the cochleas were immunolabeled as follows: Turns were washed in Tris buffered saline (TBS, pH 7.6), permeabilized by freeze/thawing in 30% sucrose^41^ using a −80 °C freezer, thawed at room temperature, washed in PBS for 20 minutes on a rotator. Tissue was blocked for 1 h in blocking buffer −5% BSA +10% Normal Donkey Serum (Jackson Immunoresearch, West Grove, PA)- in TBS containing 1% Triton X-100 (Sigma-Aldrich, now Millipore Sigma, Burlington, MA). Primary antibodies were diluted in blocking buffer and incubated overnight at 37 °C. For immunohistology (Fig. 1b), the antibodies were: for hair cells, rabbit anti-myosin-VIIa, dilution 1:200 Proteus Biosciences (Ramona, CA); for neurons, chicken anti- neurofilament H, dilution 1:200 (anti-NF-H from Millipore-Sigma, Darmstadt, Germany). After washing in PBS, samples were incubated two hours at 37 °C with secondary antibodies: AffiniPure F(ab’)_2_ Fragment Donkey-anti-Chicken IgY (IgG), AlexaFluor 488 (1:200; Jackson ImmunoResearch, West Grove, PA); and AffiniPure F(ab’)_2_ Fragment Donkey-Anti-Rabbit IgG, AlexaFluor647 (1:200, Jackson ImmunoResearch) diluted in blocking buffer with 1% Triton X-100. After PBS washes, tissues were mounted on slides with cover slips in a water soluble Fluorescence Mounting Medium (DAKO, Agilent Technologies, Santa Clara, CA). Slides were stored at 4 °C for up to three years. Slides were imaged with Nikon C2 plus scanning confocal microscope at Northwestern University Center for Advanced Microscopy. Half-turns of the cochlea were imaged using a 20x Differential Interference Contrast magnification objective lens and the images were analyzed using NIS (Nikon Imaging Software, Melville, NY). For area measurement, half-turn contours were manually drawn over an image. Then using NIS manual measurement option, the surface area data were automatically recorded using “Annotation and Measurement table” tool option and exported in excel spreadsheet.

### Genomic DNA extraction

Coverslips were removed from archival slides in saline buffer (0.1 M Tris, 0.1 M NaCl, pH 7.4) at room temperature using the blade of a scalpel. Cochlear half-turns were separated from the gel of the water soluble mounting media and transferred to a 1.5 ml micro-centrifuge tube with the scalpel blade. Archival cochlear fixed tissue as well as unfixed liver and paraformaldehyde fixed known male and female guinea pig pinna tissues were processed for genomic DNA using QIAamp DNA FFPE Tissue Kit (Qiagen, Hilden, Germany) following manufacturer recommendations, excluding the deparaffinization step and using the 90 °C decrosslinking step only for fixed tissue. Proteinase K digestion was carried out at 56 °C in a water bath and the decrosslinking step at 90 °C in a heat block filled with water. For all samples, the genomic DNA was eluted with 20 µl of Qiagen ATE elution buffer. Concentration of genomic DNA (gDNA) was determined by measuring nucleic acid absorbance at 260 nm and ratio of 260/280. Most of the samples (85%) had a 260/280 above 1.8. A few samples with a lower ratio were included and were also successful in the sex typing assay.

### PCR multiplex sex genotyping

Predicted *Cavia porcellus* Dystrophin DNA sequence (DMD-201 ENSCPOT00000009179.3; Cavpor3.0: DS562933.1:1:8140136:1 for Exon 21) and partial *Sry* gene^32^ (AJ003126.1) sequences were retrieved from Ensembl (EMBL-EBI, Hinxton, UK) and Genbank databases (NCBI, Bethesda, MD) respectively. *Dystrophin* PCR primer sequences in 5′ to 3′ direction were designed as DYS-F (GTGTTAATGGTG ACAGCATCAGC) and DYS-R (GTGCTGTTGGATCTGAAGTGGAGG) in Exon 21. Guinea pig primers for *Sry* gene were designed using partially sequenced *Sry Cavia porcellus* sequence (AJ 003126.1) and predicted *Bos taurus* (NC_016145.1), *Mus musculus* (NC_000087.7), *Rattus norvegicus* (NC_024475.1), *Homo sapiens* (NC_000024.10) and *Sus scrofa* (NC_010462.2) *Sry* sequences retrieved from Genbank. Sequence alignment was performed using MUSCLE (**MU**ltiple **S**equence **C**omparison by **L**og- **E**xpectation; EMBL-EBI, Hinxton, UK) multiple alignment tools. The designed *Sry* primer sequences were SRY-F1 (CCATGATTGCATTTATGGTGTGGTCCCG) and SRY-R1 (GCCTTTTTTCGGCTTCTGTAAGCATTTTCCAC). PCR reaction (25 µl) was performed using DreamTaq Hot Start Green PCR mix solution (Thermo Fisher Scientific, Waltham, MA), 0.4 µM of each Dystrophin primer, and 0.8 µM of each *Sry* primer and up to 20 ng of genomic DNA solution. Thermocycling PCR steps were 95 °C for 3 min followed by 36 cycles at 95 °C for 30 sec, 58 °C for 30 sec and 72 °C for 30 sec and a final cycle at 72 °C for 5 min. PCR amplicons were analyzed on 2% Agarose gels and stained with ethidium bromide. Expected amplicon sizes were 212 base pairs (bp) and 135 bp for *Dystrophin* and *Sry* gene amplification respectively. Once the PCR guinea pig product was sequenced, a second set of *Sry* PCR primers specific to guinea pig was also designed: SRY-F2 (CCGAGACCAGAGACGCAAGATAGC) and SRY-R2 (CACTGGTATCCCAGCAGCTTGC). For these primers (0.8 µM each), the annealing temperature was set at 62 °C without changing the other steps of the multiplex amplification program.

### Gel Imaging

UV gel images, originally white bands on a black background (see Supplementary Figures 1–3), were inverted to show black bands on a white background, and the pertinent regions were cropped.

### PCR amplicon sequencing

For PCR amplicon sequencing, 50 µl of PCR reactions (two PCR reactions) from un-fixed liver from two different male Guinea pigs (*Sry* PCR) from Kuiper breeder or from four fixed Kuiper and two fixed male from Charles River breeder (*Sry* PCR) and two Kuiper female (*Dystrophin* PCR) pinna were processed separately for PCR buffer removal using Wizard SV gel and PCR Clean-up System (Promega, Madison, WI), then suspended in 25 µl of DNAse-free water. Both strands of each amplicon were sequenced by the Sanger traditional method by Northwestern University Sequencing Core Facility. Deduced sequences were aligned using MUSCLE tools and compared to predicted Cavia porcellus dystrophin mRNA (DMD-201 ENSCPOT00000009179.3) for Dystrophin PCR or to generate a consensus sequence for *Sry* gene. The final amplicon consensus sequence of *Sry* partial gene was finally aligned to other species described above using MUSCLE tools (see Fig. 3).

### Data Availability Statement

All data generated or analysed during this study are included in this published article.

## Electronic supplementary material

Supplementary Information
