# Supplementary material for: Sex Genotyping of Archival Fixed and Immunolabeled Guinea Pig Cochleas
Source: Sci Rep. 2018 Mar 26;8:5156. doi: 10.1038/s41598-018-23491-3 (PMC5980087; doi:10.1038/s41598-018-23491-3)
Supplement: Supplementary file 1 — Supplementary Information [file 41598_2018_23491_MOESM1_ESM.pdf]

Supplementary Information

Sex Genotyping of Archival Fixed and Immunolabeled Guinea Pig Cochlea’s

Frédéric F. Depreux, Lyubov Czech, & Donna S. Whitlon

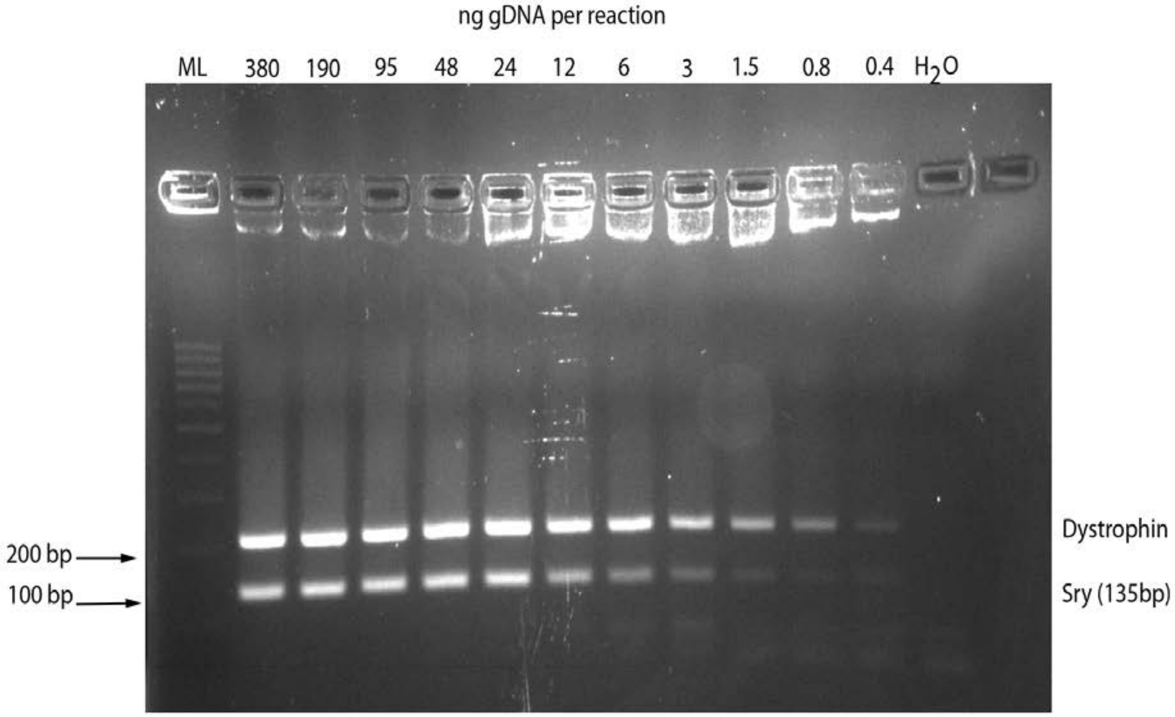

Figure S1. Full length original gel source of Figure 2a. Analytical gel resolving sex genotyping multiplex PCR of guinea pig tissue. Concentration titration of gDNA template. Representative gel (one of triplicate experiments) of PCR products from gDNA of fixed guinea pig male pinna tissue. Dysrophin and Sry PCR products are expected to run at 212 and 135 bp respectively. ML, molecular ladder

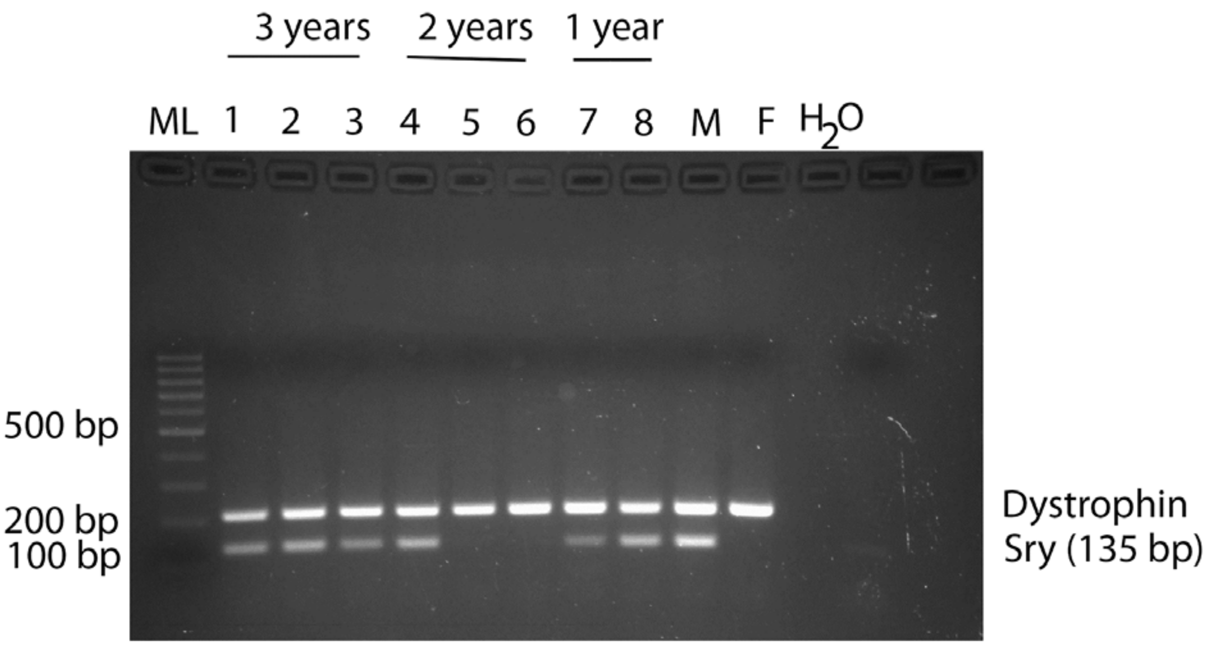

Figure S2. Full length original gel source for Figure 2b. Analytical gel resolving sex genotyping multiplex PCR of guinea pig tissue. Sex genotyping using a first deduced guinea pig Sry PCR primer set generating a 135 bp amplicon from three (lane 1-3), two (lanes 4-6) and one (lane 7-8) year old immunostained archived, cochlear half turns. ML, molecular ladder. M and F are control (known) Male and Female genomic DNA from fixed pinna extraction. Dystrophin, 212 bp.

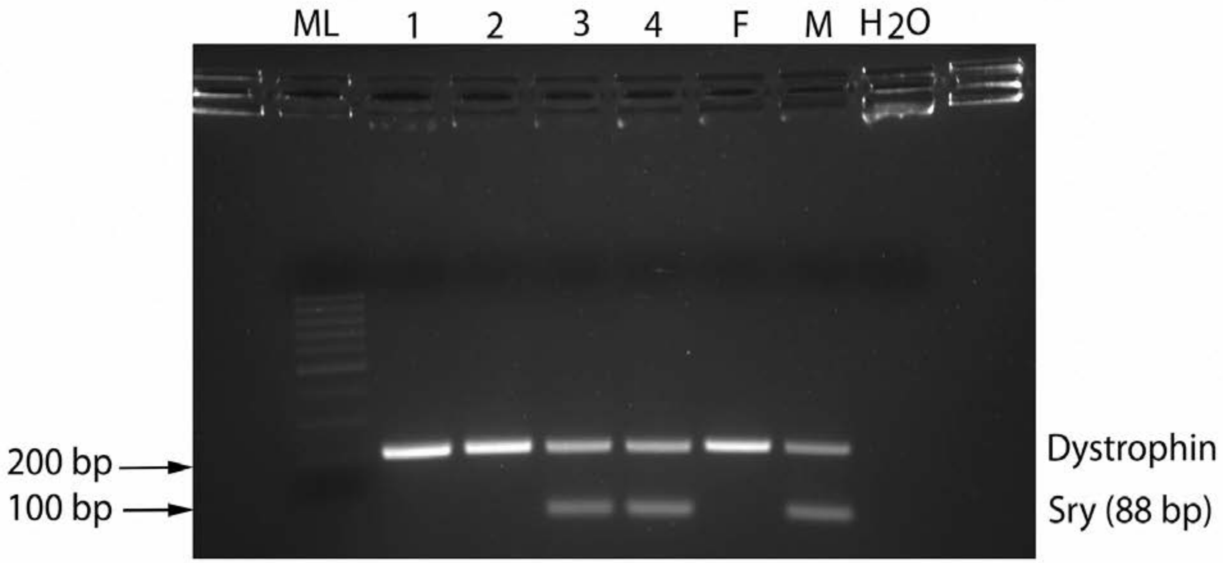

Figure S3. Full length original gel source for Figure 2c. Analytical gel resolving sex genotyping multiplex PCR of guinea pig tissue. Sex genotyping PCR using a second PCR primer set specifically derived from sequencing of the original PCR product, generates an 88 bp amplicon from one (lanes 1-2) and three (lane 3-4) year old immunolabeled, archived cochlear turns. ML, molecular ladder. M and F are control (known) Male and Female genomic DNA from fixed pinna extraction. Upper band running at 212 bp is the Dystrophin amplicon.
